# An Experimental Study to Evaluate the Antidepressant Effect of Zileuton and Aqueous Extract of Phyllanthus emblica in Unpredictable Chronic Mild Stress-Induced Depression in Albino Mice

**DOI:** 10.7759/cureus.72859

**Published:** 2024-11-01

**Authors:** Amitoj Sohal, Shirish S Joshi, Paresh G Koli, Pranali V Puradkar, Vivek C Date

**Affiliations:** 1 Pharmacology and Therapeutics, Seth Gordhandas Sunderdas Medical College and King Edward Memorial (KEM) Hospital, Mumbai, IND

**Keywords:** depression, fluoxetine, interleukin-6 (il-6), medicinal plant, phyllanthus emblica, stress, zileuton

## Abstract

Introduction: Oxidative stress and proinflammatory signaling in the brain have been found to have a significant role in the pathogenesis of depression. Therefore, drugs that reduce oxidative stress and neuroinflammation may be helpful in depression as monotherapy or as an adjunct to conventional antidepressants. Zileuton, a 5-lipoxygenase inhibitor, has been identified as a putative antidepressant in an in vitro study. It was shown to modulate the proinflammatory macrophage response in the brain and, hence, neuroinflammation. Currently, there are no antidepressants that target the inflammatory component of depression. Hence, this would be a desirable addition to our armamentarium against depression, particularly in patients with poor responses to antidepressants. *Phyllanthus emblica*, commonly known as amla, is an important medicinal plant from the traditional Indian system of medicine. It has significant antioxidant and anti-inflammatory activity and is believed to be neuroprotective. However, its role as an antidepressant needs further elucidation.

Methods: The primary aim of this study was to evaluate whether zileuton and aqueous extract of *Phyllanthus emblica* exert antidepressant activity in a chronic stress model of depression in Swiss albino mice. A total of six groups (six mice each) were used in the study. They were disease control (given normal saline), positive control (PC; given fluoxetine), *Phyllanthus emblica* low dose and high dose, and zileuton low and high dose. After induction of depression, drugs were given for 14 days. The antidepressant effect of the study drugs was evaluated using two behavioral tests: the tail suspension test (TST) and the forced swim test (FST). Interleukin-6 (IL-6) levels in the hippocampus were measured on day 45. Descriptive statistics and ANOVA were used for the statistical analysis. P-value < 0.05 was considered statistically significant.

Results: It was observed that zileuton and aqueous extract of *Phyllanthus emblica *decreased the duration of immobility in both behavioral tests, which indicated their antidepressant effect. Also, it was observed that the antidepressant effect of zileuton and *Phyllanthus emblica* was comparable to fluoxetine. There was a statistically significant decrease (p < 0.001) in hippocampal IL-6 levels in the positive control (fluoxetine) group, *Phyllanthus emblica* low dose and high dose groups, and zileuton low dose and high dose groups compared to the disease control group.

Conclusion: According to our study's findings, zileuton and *Phyllanthus emblica* exert an antidepressant effect comparable to that of fluoxetine, as evidenced by the reduction in immobility time in the behavioral tests. The reduction in the IL-6 levels by zileuton and *Phyllanthus emblica* signifies a decrease in neuroinflammation, which may be responsible for the antidepressant effect.

## Introduction

Depression is the most common mental health disorder, severely impacting the quality of life [1]. Major depressive disorder (MDD), also known as clinical depression, is characterized by at least two weeks of pervasive low mood, low self-esteem, and loss of interest or pleasure in normally enjoyable activities and significantly affects your daily life. It is estimated that over 300 million people in the world have depression [2]. A World Health Organization (WHO) report predicted that MDD would become the leading cause of disability in the world by 2030 [3].

Despite remarkable progress, no single model or mechanism can satisfactorily explain all aspects of MDD. First-line pharmacological treatment for MDD addresses monoamine imbalance with selective serotonin reuptake inhibitors (SSRIs) or serotonin-norepinephrine reuptake inhibitors (SNRIs), with responses achieved in only one-third of the patients [4]. There is an unmet need for novel drugs with a faster onset, fewer side effects, and higher remission rates, leveraging alternate targets and mechanisms of action.

Recent insights into the pathophysiology of depression have revealed that oxidative stress (OS) and proinflammatory signaling are major factors in the pathogenesis of depression, leading to mitochondrial dysfunction, which results in impaired neuronal and synaptic plasticity and decreased neurogenesis. It is well established that patients with MDD have an imbalance in the levels of reactive oxygen species (ROS) versus the anti-oxidant mechanisms. Patients with depression also have higher plasma levels of proinflammatory cytokines [5,6]. Therefore, targeting these changes with suitable drugs having anti-inflammatory/anti-oxidant activity or both could be an effective strategy for treating depression [5].

Zileuton, which is currently approved for the maintenance treatment of asthma, is a 5-lipoxygenase inhibitor. It inhibits the synthesis of leukotrienes, an integral part of the inflammatory cascade, inhibiting an inflammatory response. A study using in vitro and in silico approaches identified zileuton as a putative antidepressant, meriting further elucidation [7]. *Phyllanthus emblica*, commonly known as the Indian gooseberry or amla, is an essential medicinal plant in the traditional Indian system of medicine with extensive historical usage for preventing memory loss [8]. Its fruit extracts contain high amounts of vitamin C and many phytoconstituents, such as polyphenols, flavonoids, and tannins. These phytoconstituents impart antioxidant and anti-inflammatory activities to the extract, making them beneficial for diversified ailments and possibly depression [9]. The current study was undertaken to evaluate the antidepressant effect of these drugs (zileuton and aqueous extract of *Phyllanthus emblica*) in unpredictable chronic mild stress (UCMS)-induced depression in albino mice using fluoxetine as a positive control and to assess the effect of both drugs on the hippocampal interleukin-6 (IL-6) levels (a marker of neuroinflammation) [10,11]. The model involves exposing the animal to minor stressors at unpredictable intervals for several weeks. This results in several behavioral alterations in most animals (some animals can be more stress-resistant), including anhedonia (loss of pleasure) and apathy.

This article was previously presented as an oral paper presentation at NAPTICON 2022 on October 28, 2022, and as a poster presentation at IntPCON 2022 on December 9, 2022.

## Materials and methods

The Institutional Animal Ethics Committee, Seth G.S. Medical College and K.E.M. Hospital (IAEC/GSMC/05/2020) gave permission before the study began. The study was conducted between 8 November 2021 and 31 August 2022, following the Committee for Control and Supervision of Experiments on Animals (CCSEA) recommendations and funded by a competitive intramural grant.

The animals were bred at random in an institutional facility. A total of 48 Swiss albino mice of any gender weighing 25-35 grams were used and randomly assigned to the study groups. The mice were housed in the experimental area seven days before the initiation of the experiment to allow for acclimatization. Twelve of the 48 mice were used in phase 1 (standardization of the model), while the remaining were employed in phase 2. Animals were housed in polypropylene cages with stainless steel top grills and facilities to provide food and water. Paddy husk was used as the bedding. Animals were housed in a controlled environment with a temperature range between 23°C ± 4°C, relative humidity of 30-70%, and a 12-hour light-dark cycle. Animals were housed under standard laboratory conditions, with free access to filtered water and commercial animal feed in pellets.

The test drugs zileuton and fluoxetine were bought from Sri Hari Fine Chem, Mumbai, India, and the aqueous extract of *Phyllanthus emblica* was procured from Dhootapapeshwar Ayurvedic Research Foundation, Navi Mumbai, India. GENLISA® ELISA (enzyme-linked immunosorbent assay) kits were purchased from KRISHGEN BioSystems (Mumbai, India) to estimate the hippocampal IL-6 levels, and the levels were determined using the sandwich ELISA technique.

**Figure 1:**
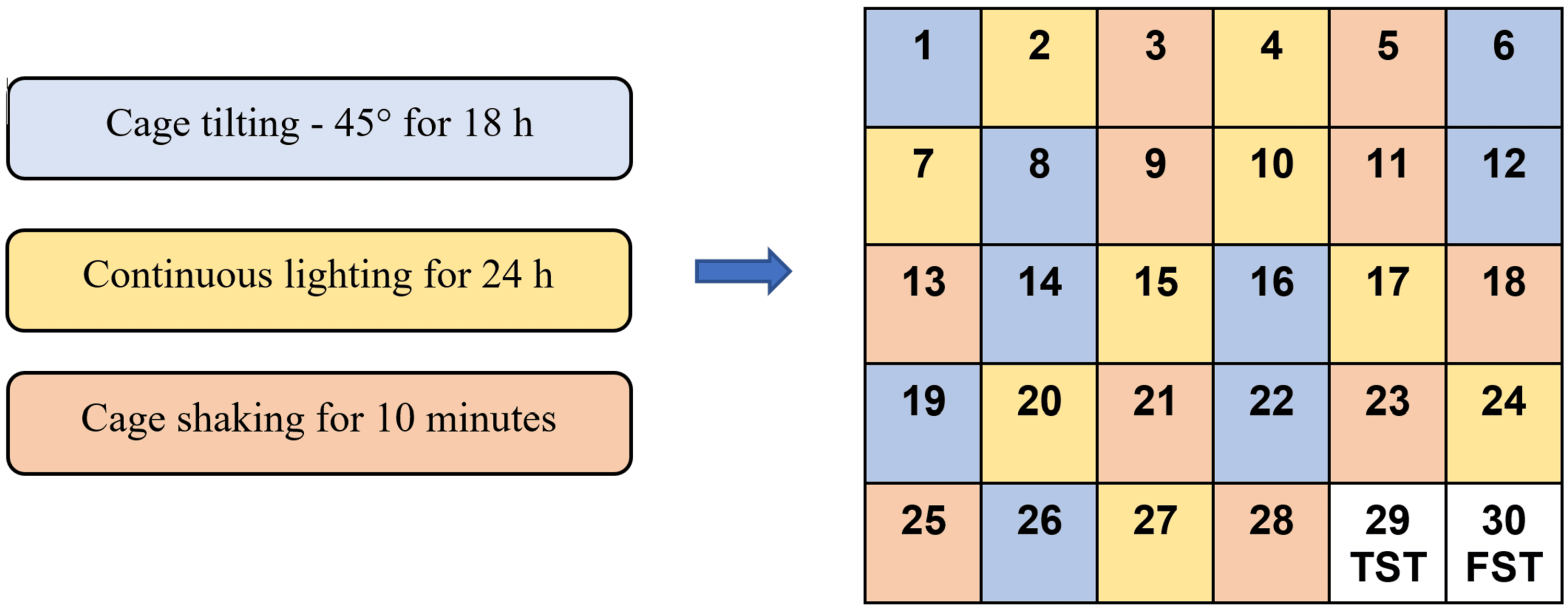
Protocol for unpredictable chronic mild stress model followed by behavioral tests (phase 1). Each box represents a day. TST: tail suspension test; FST: forced swim test.

UCMS-induced depression in albino mice was used for the study. Before the commencement of drug administration, the UCMS model was standardized in our institution for 28 days (Figure 1). This widely used UCMS model involves unpredictably subjecting animals to various mild stressors over four weeks [12,13]. The stressors included 45° cage tilting for 18 hours, continuous lighting for 24 hours, and cage shaking for 10 minutes. The same stress was never given on two successive days. After administering stressors for 28 days, the mice were subjected to the tail suspension test (TST) and forced swim test (FST).

Behavioral tests

Tail Suspension Test

A sticking tape was applied over the tail, with both ends fixed to the table's edge at 58 centimeters. Each mouse was left in this position for five minutes, and the time it remained immobile was noted [14].

Forced Swim Test

A plexiglass cylinder with a height of 25 centimeters and a diameter of 12 centimeters was filled with water up to a height of 15 centimeters (Figure 1). The water temperature was kept at 25 ± 3°C. The mice were forced to swim, one at a time, inside the plexiglass cylinder. The duration of immobility over an observation period of five minutes was noted [15].

The duration of immobility was measured for both tests and compared between the normal control group versus the disease control group. Table 1 shows the number of animals per group with the procedure followed for induction.

**Table 1:** Distribution of animals for standardization of unpredictable chronic mild stress model (phase 1).

S. No.	Group	Procedure	Number of Swiss albino mice
1	Normal control	No stressors	6
2	Disease control	Unpredictable chronic mild stress as per the protocol	6

Phase 2 was carried out using 36 mice given stressors according to phase 1 (same number of days). The stressors were given for 28 days for induction and continued for another 14 days during the drug treatment. Following induction, the 36 mice were divided into six equal groups and administered drugs orally for 14 days (one dose per day) as per the allotted group. The experimental groups and drugs administered for phase 2 are described below (Table 2).

**Table 2:** Groups of animals for phase 2.

Sr. No.	Groups (N = 6)	Drug given for 14 days
1	Disease control (DC)	Vehicle control: normal saline (0.5 ml p.o.)
2	Positive control (PC)	Fluoxetine (20 mg/kg p.o.) [16]
3	*Phyllanthus emblica* low dose (PE1)	Aqueous extract of *Phyllanthus emblica* (1560 mg/kg p.o.) [16]
4	*Phyllanthus emblica* high dose (PE2)	Aqueous extract of *Phyllanthus emblica* (3120 mg/kg p.o.) [16]
5	Zileuton low dose (Zil 1)	Zileuton (50 mg/kg p.o.) [17]
6	Zileuton high dose (Zil 2)	Zileuton (100 mg/kg p.o.) [17]

**Figure 2:**
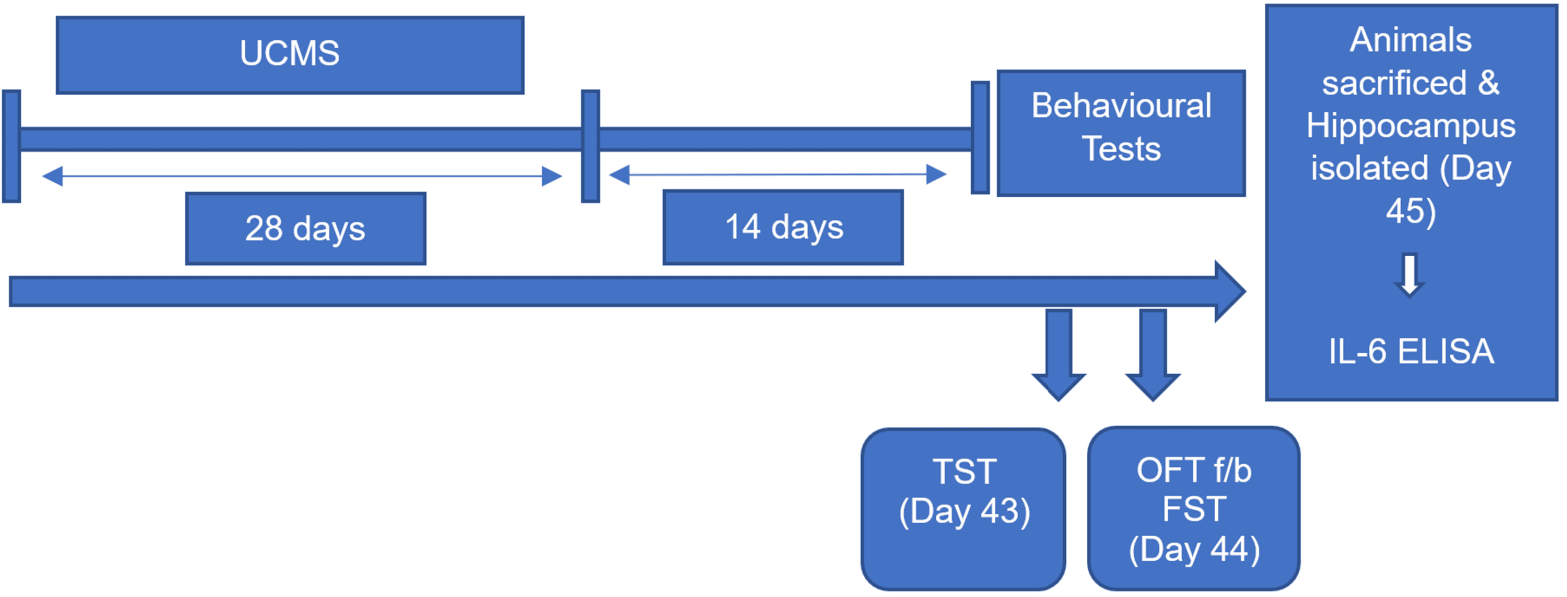
Schematic representation of the phase 2. UCMS: unpredictable chronic mild stress; TST: tail suspension test; FST: forced swim test; OFT: open field test; f/b: followed by; IL-6: interleukin-6; ELISA: enzyme-linked immunosorbent assay.

The antidepressant effect of study drugs was evaluated using the behavioral tests TST and FST. An open field test (OFT) was conducted (to check the number of zone entries) five minutes before subjecting the mice to FST to rule out the test drugs' nonspecific stimulant effect. Each mouse was individually placed in the center of the apparatus. Locomotory activity (number of zone entries) was recorded for five minutes using the Mazemaster video tracking software (VJ Instruments, Karanja Lad, India). The animals were sacrificed on day 45, and the hippocampal IL-6 levels were measured using ELISA (Figure 2).

Interleukin-6 estimation (hippocampus)

Following behavioral tests, the animals were sacrificed using ketamine on day 45. Bilateral hippocampi were removed after brain dissection and rinsed in phosphate buffered saline (PBS; pH 7.4) to remove the excess blood. The hippocampi were weighed, and PBS was added according to the weight of the tissue. The tissues were then homogenized. The homogenate was then centrifuged at 2000 RPM for 20 minutes. The supernatant was collected and stored at -20°C. The IL-6 levels in the stored samples were estimated using ELISA.

Statistical analysis

Data were analyzed using SPSS version 26 (IBM Corp., Armonk, NY). Data normality distribution was tested using the Shapiro-Wilk test, and appropriate transformations were performed if required. Comparisons between groups were performed using the unpaired t-test (phase 1). Parametric data were analyzed using one-way ANOVA followed by post-hoc Tukey’s (phase 2) test. The level of significance was set at p < 0.05.

## Results

Results of phase 1

**Figure 3:**
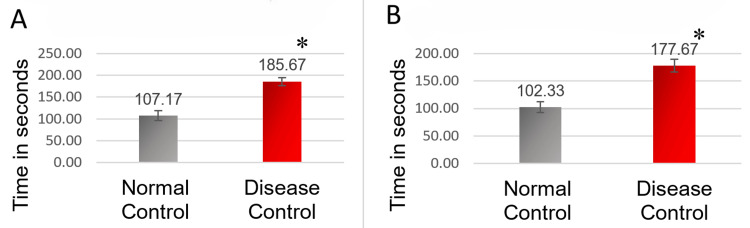
Graphical representation of results of standardization (n = 6/group). “*” represents p < 0.001 versus the normal control group, using an unpaired t-test. A: Duration of immobility in tail suspension test. B: Duration of immobility in forced swim test.

The standardization procedure was performed to establish the UCMS model at our institution. TST and FST were performed on day 29 and day 30, respectively, as described in the section above. We observed a statistically significant increase in the duration of immobility on performing the TST and FST (p < 0.001) in the disease control group (UCMS group) compared to normal control (no stressors). Figure 3 summarizes the results of standardization.

Results of phase 2

**Figure 4:**
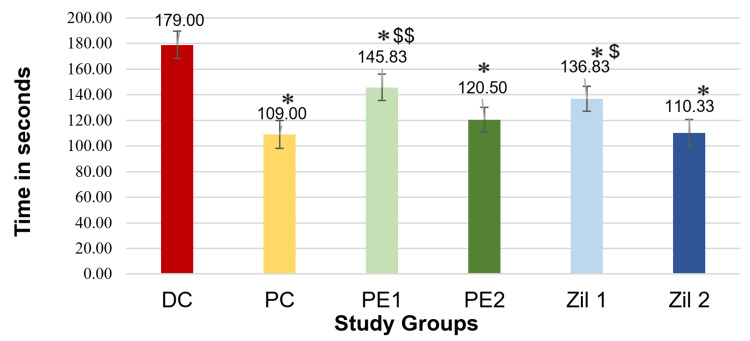
Graphical representation of results of the duration of immobility in tail suspension test (phase 2) (n = 6/group). * P < 0.001 vs. disease control group; $ p < 0.01 and $$ p < 0.001 vs. positive control group; one-way ANOVA and post hoc Tukey’s test. DC: disease control; PC: positive control; PE1: *Phyllanthus emblica* low dose; PE2: *Phyllanthus emblica* high dose; Zil 1: zileuton low dose; Zil 2: zileuton high dose.

Figure 4 depicts the values obtained from the TST represented as mean and standard deviations. Application of one-way ANOVA followed by post hoc Tukey's test showed a statistically significant decrease (p < 0.001) in the duration of immobility in the positive control (PC; fluoxetine) group, *Phyllanthus emblica* low dose and high dose groups (PE1 and PE2), and zileuton low dose and high dose groups (Zil 1 and Zil 2) compared to disease control. Additionally, we found no statistically significant difference in the duration of immobility between the PE2 group and Zil 2 group (higher dose of *Phyllanthus emblica* and zileuton) when compared to the positive control group (fluoxetine), indicating that these groups were comparable.

**Figure 5:**
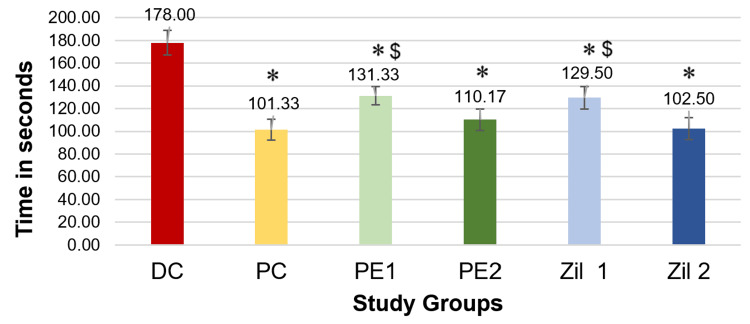
Graphical representation of results of the duration of immobility in forced swim test (phase 2) (n = 6/group). * P < 0.001 vs. disease control group, $ p < 0.001 vs. positive control group; one-way ANOVA and post hoc Tukey’s test. DC: disease control; PC: positive control; PE1: *Phyllanthus emblica* low dose; PE2: *Phyllanthus emblica* high dose; Zil 1: zileuton low dose; Zil 2: zileuton high dose.

Similarly, Figure 5 depicts the immobility time when conducting FST. Pre-testing with OFT revealed no statistically significant differences (Table 3). Application of one-way ANOVA followed by post hoc Tukey's test showed a statistically significant decrease (p < 0.001) in the duration of immobility in the PC (fluoxetine) group, *Phyllanthus emblica* low dose and high dose groups (PE1 and PE2), and Zileuton low dose and high dose groups (Zil 1 and Zil 2) compared to the disease control group. Additionally, the duration of immobility in the PE2 group and Zil 2 group was comparable to that of the PC group.

**Table 3:** Results of the open field test. Compared by one-way ANOVA.

Groups (n = 6/group)	Total number of zone entries (mean ± SD)	P-value
Disease control (DC)	97.67 ± 12.44	
Positive control (PC)	102.00 ± 15.48	>0.05 vs. all groups
*Phyllanthus emblica* low dose (PE1)	98.67 ± 15.03	>0.05 vs. all groups
*Phyllanthus emblica* high dose (PE2)	102.50 ± 9.52	>0.05 vs. all groups
Zileuton low dose (Zil 1)	97.00 ± 9.14	>0.05 vs. all groups
Zileuton high dose (Zil 2)	101.00 ± 13.40	>0.05 vs. all groups

Hippocampal interleukin levels

**Figure 6:**
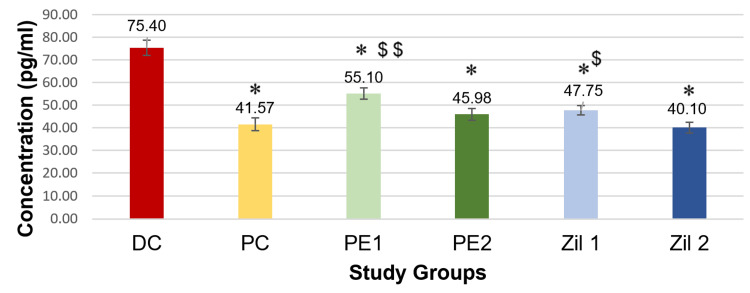
Graphical representation of hippocampal interleukin-6 levels (phase 2) (n = 6/group). * P < 0.001 vs. disease control group; $ p < 0.01 and $$ p < 0.001 vs. positive control group; ANOVA and post hoc Tukey’s test. DC: disease control; PC: positive control; PE1: *Phyllanthus emblica* low dose; PE2: *Phyllanthus emblica* high dose; Zil 1: zileuton low dose; Zil 2: zileuton high dose.

There was a statistically significant decrease (p < 0.001) in hippocampal IL-6 levels in the PC (fluoxetine) group, *Phyllanthus emblica* low dose and high dose groups (PE1 and PE2), and zileuton low dose and high dose groups (Zil 1 and Zil 2) compared to the disease control group on the application of one-way ANOVA followed by post hoc Tukey's test. Furthermore, the IL-6 levels in the PE2 and the Zil 2 groups were comparable to the PC group, with the lowest levels in the zileuton 100 mg/kg (Figure 6).

## Discussion

Depression is a chronic and debilitating condition that has a severe negative impact on psychosocial functioning and quality of life [3]. There is a pressing need to identify novel drugs with alternate mechanistic targets beyond the monoamine pathways, which led to the initiation of the current study.

In the current study, the zileuton and *Phyllanthus emblica*-treated groups showed a significant decrease in the duration of immobility in the behavioral tests compared to the disease control group. Thus, the results of the behavioral tests showed that both *Phyllanthus emblica* and zileuton exert an antidepressant effect. The antidepressant effect of the higher *Phyllanthus emblica* and zileuton dose was comparable to fluoxetine. Both zileuton and *Phyllanthus emblica* significantly lowered the hippocampal IL-6 levels, which may have contributed to the antidepressant effect observed in the behavioral tests. The efficacy shown in the study makes a compelling case for investigation in extensive pre-clinical studies to facilitate drug development and repurposing. The supportive biomarker analysis showing an association with reduced levels of hippocampal IL-6 by administration of these drugs is a novel finding that has not been previously reported in the literature. Our results confirm the findings in published literature.

In a study conducted by Li et al., zileuton was shown to ease depressive-like behaviors in lipopolysaccharide (LPS)-induced depression in mice. Mice treated with zileuton (50 mg/kg and 100 mg/kg) showed significantly reduced immobility time in the TST and FST compared to the LPS-treated disease control group [18]. In our study, zileuton (at doses of 50 mg/kg and 100 mg/kg) was also found to significantly reduce the duration of immobility in the TST and FST in a more robust model of depression.

Another study inducing depression using the chronic restraint stress (CRS) model showed that treatment with zileuton (50 mg/kg, 100 mg/kg) significantly decreased the immobility time in the behavioral tests. The results also showed weakened nuclear factor-ƙβ signaling and an inflammatory response on western blot [17]. Our study also showed that zileuton reduced hippocampal inflammation, as evidenced by the significant reduction in the IL-6 level, a pro-inflammatory cytokine implicated in depression.

The findings about *Phyllanthus emblica* reduced immobility time on behavioral tests and comparable activity to fluoxetine are also supported by published literature. A study was conducted to evaluate the antidepressant potential of *Phyllanthus emblica* on acute and chronic administration. For the acute study, a single dose of the study drugs was given one hour before conducting the behavioral tests. The study drugs were administered for 10 days for the chronic study, and the last dose was administered one hour before conducting the behavioral tests. In both the acute and chronic studies, *Phyllanthus emblica* showed a more significant decrease in the duration of immobility than imipramine (positive control) [19]. In our study, using fluoxetine as the positive control, the duration of immobility in the *Phyllanthus emblica* (high dose) group was comparable to fluoxetine.

Another study conducted to evaluate the antidepressant activity of *Phyllanthus emblica* using the TST and FST showed that at higher doses (1560 mg/kg and 3120 mg/kg), aqueous extract of *Phyllanthus emblica* significantly reduced the immobility time compared to the control group [16]. Our study also showed a significant reduction in the duration of immobility in both the TST and FST compared to the disease control group with these doses.

In our study, we induced depression using the UCMS model, followed by the administration of aqueous extract of *Phyllanthus emblica* for 14 days. In the behavioral tests conducted thereafter, the duration of immobility in the *Phyllanthus emblica*-treated group (high dose) was comparable to the positive control group (fluoxetine 20 mg/kg). Hence, our study also found that the antidepressant activity of the higher dose of the aqueous extract of *Phyllanthus emblica* was comparable to fluoxetine (20 mg/kg) in a more robust four-week-long UCMS model of depression.

The study's strengths lie in it being the first study with the study drug and novel findings about biomarker analysis (IL-6) and using two behavioral tests to reduce bias. The study has the limitation of not analyzing multiple other markers of neuroinflammation and neurogenesis (e.g., brain-derived neurotrophic factor - BDNF) due to logistical constraints. The opportunities arising out of the study are several, particularly regarding *Phyllanthus emblica*, which showed efficacy at high doses comparable to fluoxetine and may represent a viable alternative for combination therapy or replacement therapy in the setting of significant side effects precluding fluoxetine usage or non-alleviation of symptoms on fluoxetine therapy. The neuroprotectant and neuro-regenerative properties need further elucidation in pre-clinical studies with robust multiple biomarker analysis, given the possible scope of expansion of usage in the setting of depressive disorders associated with neurological disorders. Furthermore, currently, there are no antidepressants that target the inflammatory component of depression. Zileuton, upon further evaluation, may prove to be a desirable addition to our armamentarium against depression, particularly in patients with poor response to the available antidepressants.

## Conclusions

According to our study, zileuton and *Phyllanthus emblica* exert an antidepressant effect comparable to fluoxetine, as evidenced by the reduction in immobility time in the behavioral tests. The reduction in the IL-6 levels by zileuton and *Phyllanthus emblica* signifies a decrease in neuroinflammation, which may be responsible for the antidepressant effect. *Phyllanthus emblica* is comparable to zileuton in our study, and it needs to be further investigated to find its effectiveness in humans.
